# Drug-Resistant Tuberculosis among HIV-Infected Patients Starting Antiretroviral Therapy in Durban, South Africa

**DOI:** 10.1371/journal.pone.0043281

**Published:** 2012-08-17

**Authors:** Jeffrey K. Hom, Bingxia Wang, Senica Chetty, Janet Giddy, Matilda Mazibuko, Jenny Allen, Rochelle P. Walensky, Elena Losina, Kenneth A. Freedberg, Ingrid V. Bassett

**Affiliations:** 1 Harvard Medical School, Boston, Massachusetts, United States of America; 2 Division of General Medicine, Massachusetts General Hospital, Boston, Massachusetts, United States of America; 3 McCord Hospital, Durban, South Africa; 4 University of KwaZulu-Natal Microbiology Laboratory and the Medical Research Council, Durban, South Africa; 5 Division of Infectious Disease, Massachusetts General Hospital, Boston, Massachusetts, United States of America; 6 Medical Practice Evaluation Center, Massachusetts General Hospital, Boston, Massachusetts, United States of America; 7 Department of Biostatistics, Boston University School of Public Health, Boston, Massachusetts, United States of America; 8 Department of Epidemiology, Boston University School of Public Health, Boston, Massachusetts, United States of America; 9 Division of Infectious Disease, Brigham and Women’s Hospital, Boston, Massachusetts, United States of America; 10 Department of Orthopedics, Brigham and Women’s Hospital, Boston, Massachusetts, United States of America; 11 Harvard Center for AIDS Research (CFAR), Harvard University, Boston, Massachusetts, United States of America; Institute of Infectious Diseases and Molecular Medicine, South Africa

## Abstract

**Objective:**

To estimate the prevalence of drug-resistant tuberculosis (TB) and describe the resistance patterns in patients commencing antiretroviral therapy (ART) in an HIV clinic in Durban, South Africa.

**Design:**

Cross-sectional cohort study.

**Methods:**

Consecutive HIV-infected adults (≥18y/o) initiating HIV care were enrolled from May 2007–May 2008, regardless of signs or symptoms of active TB. Prior TB history and current TB treatment status were self-reported. Subjects expectorated sputum for culture (MGIT liquid and 7H11 solid medium). Positive cultures were tested for susceptibility to first- and second-line anti-tuberculous drugs. The prevalence of drug-resistant TB, stratified by prior TB history and current TB treatment status, was assessed.

**Results:**

1,035 subjects had complete culture results. Median CD4 count was 92/µl (IQR 42–150/µl). 267 subjects (26%) reported a prior history of TB and 210 (20%) were receiving TB treatment at enrollment; 191 (18%) subjects had positive sputum cultures, among whom the estimated prevalence of resistance to any antituberculous drug was 7.4% (95% CI 4.0–12.4). Among those with prior TB, the prevalence of resistance was 15.4% (95% CI 5.9–30.5) compared to 5.2% (95% CI 2.1–8.9) among those with no prior TB. 5.1% (95% CI 2.4–9.5) had rifampin or rifampin plus INH resistance.

**Conclusions:**

The prevalence of TB resistance to at least one drug was 7.4% among adults with positive TB cultures initiating ART in Durban, South Africa, with 5.1% having rifampin or rifampin plus INH resistance. Improved tools for diagnosing TB and drug resistance are urgently needed in areas of high HIV/TB prevalence.

## Introduction

Infection with tuberculosis (TB) remains the leading cause of mortality among HIV-infected people worldwide [1]. South Africa has the third highest TB incidence in the world, with ∼1,000 cases per 100,000 people per year and the most HIV-infected citizens of any country [2], [3]. Even for people successfully diagnosed with TB in South Africa, TB treatment completion remains a challenge. In 2005, KwaZulu-Natal, the South African province with the highest HIV prevalence, reported the greatest number of smear-positive pulmonary TB cases in the country and had the lowest cure rate (45%) [4]. The emergence of drug-resistant TB, which is challenging to diagnose and treat, threatens to undo the progress made in HIV treatment and care within an overburdened public health infrastructure in South Africa [5], [6].

In 2005, a substantial burden of multi-drug (MDR) and extensively-drug resistant (XDR) TB was identified among HIV-infected patients in the rural Msinga District of KwaZulu-Natal. Enhanced surveillance there revealed 39% MDR TB and 6% XDR TB among HIV-infected patients with culture-confirmed TB, exceeding previous surveys [7]. However, province-wide surveillance in KwaZulu-Natal has largely reflected sputum cultures performed on request based on clinical suspicion of treatment failure. This practice has varied widely among districts, with an estimated 80% of reported cases lacking culture confirmation and drug susceptibility testing [8], [9]. In addition, individual-level data, such as prior TB treatment exposure and HIV status, have not been available in population-based surveys of TB drug resistance.

In the setting of an intensive TB screening program using mycobacterial culture and drug susceptibility testing for all patients, our objective was to assess the prevalence of drug-resistant TB and to describe the resistance patterns in TB culture positive patients commencing ART in an urban HIV clinic in Durban, South Africa.

## Methods

We evaluated disease prevalence and drug resistance patterns through an intensive TB screening program for HIV-infected patients starting antiretroviral therapy in Durban, South Africa. The Sinikithemba HIV clinic, based at McCord Hospital, a semi-private urban hospital in Durban, has been offering ART since 1999. McCord Hospital is a 142-bed state-aided (public/private partnership) general hospital that serves a mainly urban population from the greater Durban area, as well as more distant parts of KwaZulu-Natal. In 2004, the clinic became a U.S. President’s Emergency Plan for AIDS Relief-funded site and subsequently initiated ART in over 8,000 patients. During the study period, the clinic provided ART literacy training to HIV-infected patients with CD4 counts <200/µl, those who met clinical criteria (WHO stage 3 or 4 disease), and patients who were transferring their care to Sinikithemba [10]. McCord charges subsidized fees for services; during the study period, patients paid an all-inclusive 90 ZAR (12 USD 2008) monthly fee for HIV care. Patients diagnosed with TB at Sinikithemba were offered TB treatment on-site, with fixed-dose regimens according to South African treatment guidelines [11].

The enrollment procedure for the study population has been previously described [12]. Briefly, for one year beginning in May 2007, a convenience sample of consecutive HIV-infected adults (≥18 years) attending ART literacy training were offered enrollment into this prospective cohort study, regardless of signs or symptoms of active TB. Patients taking TB treatment at enrollment and/or already taking ART who were newly transferring their care to Sinikithemba were also eligible. A trained research nurse obtained written consent, and then administered a 12-item questionnaire that included demographic data as well as self-reported history of prior TB infection and current TB treatment status. All patients expectorated a single sputum specimen, either spontaneously or using ultrasonic nebulization if needed, with single-use tubing to avoid contamination Samples were transported daily to the collaborative TB laboratory of the University of KwaZulu-Natal and the South African Medical Research Council in Durban.

Sputum specimens were processed for *Mycobacterium tuberculosis* culture (Middlebrook 7H11 solid agar medium and BACTEC mycobacterial growth indicator tube [MGIT] 960 System; BD). TB was confirmed in positive solid or MGIT liquid cultures containing acid fast bacilli using niacin and nitrate testing. Positive cultures were subcultured on quadrant 7H10 agar for susceptibility testing using the 1% proportional method. Isolates were tested for susceptibility to isoniazid, rifampin, streptomycin, ethambutol, ofloxacin, and kanamycin. Handling of sputum samples adhered to good microbiological practice with all sample manipulation being performed in a class II biosafety cabinet. Manipulation of each sample was completed before the next sample was manipulated. Sputum samples were decontaminated and processed in “batches” of a maximum of 10 samples, plus two freshly created standardised controls (H37Rv - positive and E.coli -negative) as well as reagent controls with each batch. Ten percent of all smears were quality controlled, and all smear-negative samples were checked by a second microscopist. A drug susceptible strain (H37Rv) and a drug resistant strain (A169) were included with each batch of susceptibilities and with each new media manufacture. All drug resistant results were confirmed by a second reader and were retested to confirm the resistance pattern. We evaluated the prevalence of drug-resistant TB, stratified by prior TB history and current TB treatment status, with 95% confidence intervals calculated using the binomial distribution.

The study was approved by the McCord Hospital Research Ethics Committee (Durban, South Africa) and the Partners Human Research Committee (protocol 2007-P-000228; Boston, Massachusetts, USA).

## Results

One thousand thirty-five patients were enrolled for whom sputum culture was carried out, representing approximately 90% of patients commencing ART literacy training during the study period. The median CD4 count was 92/µl (IQR 42–150/µl). Nebulized sputum induction was required in 167 patients (16%). Two-hundred sixty-seven patients (26%) reported a prior history of TB and 210 patients (20%) were receiving TB treatment at the time of enrollment. Forty-seven (22%) of those receiving TB treatment at enrollment reported a prior history of TB.

Sputum cultures were positive for *M. tuberculosis* in 191 (18%) patients, of which 175 (92%) had susceptibility data available. Patients with TB resistant to any drug (n = 13) were similar to patients with fully susceptible positive TB sputum cultures (N = 178) with regards to age, gender, employment status, previous hospitalization, household members with TB, and stage of HIV disease (Table 1). The median CD4 count among patients with TB resistant to any drug at the time of study enrollment (48 cells/µl, IQR 26–82 cells/µl) was lower than the median for patients with fully susceptible TB (79 cells/µl, IQR 41–131 cells/µl; p = 0.06). Forty-six percent of patients with drug-resistant TB reported a prior history of TB, compared with 22% of patients with fully susceptible TB (p = 0.05).

**Table 1:** Baseline characteristics of all patients with tuberculosis and patients with tuberculosis resistant to any drug, according to culture and sensitivity testing, in an HIV clinic in Durban, South Africa, 2007–2008.

Characteristic	Patients with tuberculosis,total (N = 191)	Patients with drug susceptible tuberculosis† (N = 178)	Patients with tuberculosis resistant to any drug (N = 13)
Demographic characteristics (%)			
Female	99 (52)	92 (52)	7 (54)
Age, median (years)	36 (31–43)	36 (31–43)	43 (32–45)
Employed	104 (55)	98 (56)	6 (46)
HIV-related characteristics (%)			
Baseline CD4 count, median cells/µl (IQR)	78 (40–125)	79 (41–131)	48 (26–82)
WHO stage 3 or 4 disease	136 (84)	127 (84)	9 (90)
On ART at study entry*	17 (9)	16 (9)	1 (8)
Other clinical characteristics (%)			
Prior history of tuberculosis	46 (24)	40 (22)	6 (46)
Household member with history of tuberculosis	43 (23)	39 (22)	4 (31)
Hospitalized in previous 5 years	38 (23)	36 (24)	2 (18)

* A small proportion of patients undergoing HIV literacy training had previously started ART.

† Includes patients missing drug susceptibility data and with partial susceptibility data without evidence of resistance.

**Figure 1:**
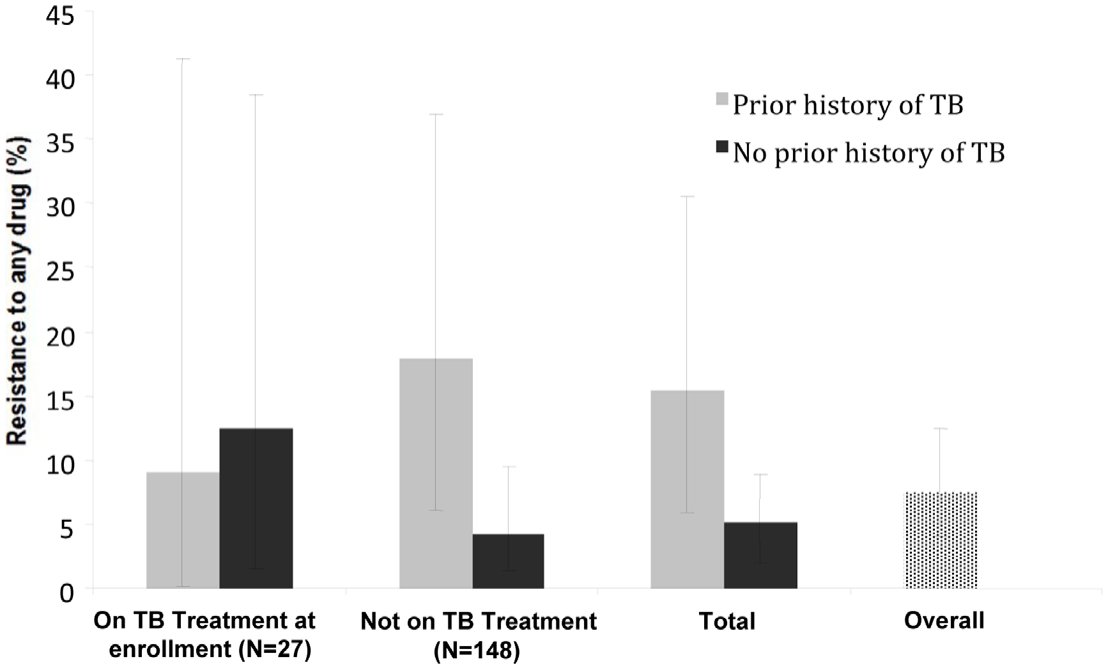
Prevalence of resistance to any drug (%), stratified by treatment status. Error bars represent 95% confidence intervals.

The prevalence of resistance to any anti-tuberculous drug was 7.4% (95% CI 4.0–12.4; Figure 1, speckled bar). Among patients on TB treatment at enrollment, the prevalence of drug-resistant TB was 9.1% (95% CI 0.2–41.3) in those with prior history of TB compared to 12.5% (95% CI 1.5–38.4) in those reporting no prior TB (p = 0.78). In patients not on TB treatment at enrollment, the prevalence of drug-resistant TB was 17.9% (95% CI 6.1–36.9) in those with prior TB versus 4.2% (95% CI 1.4–9.5) in those with no prior TB (p = 0.009). In total, among those with prior history of TB treatment, the prevalence of drug resistance was 15.4% (95% CI 5.9–30.5), compared to 5.2% (95% CI 2.1–10.3) among those with no prior history of TB (p = 0.032).

The overall prevalence of rifampin or rifampin plus isoniazid resistance (multi-drug resistant or MDR-TB) was 5.1% (95% CI 2.4–9.5). Among patients receiving TB treatment at enrollment, the prevalence of rifampin or MDR-TB was 9.1% (95% CI 0.2–41.3) in those with prior TB compared to 12.5% (95% CI 1.6–38.4) in those reporting no prior TB (p = 0.78). In patients not receiving TB treatment at enrollment, the prevalence of rifampin or MDR-TB was 10.7% (95% CI 2.3–28.2) in those with prior TB versus 2.5% (95% CI 0.5–7.1) in those with no prior TB (p = 0.04). Among those with prior history of TB treatment, the prevalence of rifampin or MDR-TB was 10.3% (95% CI 2.9–24.2), compared to 3.7% (95% CI 1.2–8.4) in those with no prior history of TB (p = 0.10).

The overall prevalence of streptomycin resistance was 2.9% (95% CI 0.9–6.5). Subjects with a prior history of TB had a 10.3% (95% CI 2.9–24.2) prevalence of streptomycin resistance, compared to 0.7% (95% CI 0.02–4.0) among subjects without a prior TB history (p = 0.002).

Two subjects had ethambutol resistance, in combination with resistance to isoniazid, rifampin, and streptomycin. One subject without prior history of TB had resistance to kanamycin alone. No ofloxacin resistance was detected.

Results of sensitivities to first- and second-line anti-TB drugs were not available for all sputum cultures. Of the 191 patients with positive cultures, 2 had partial sensitivity data and 16 had no sensitivity data available. Compared to the 173 patients with complete sensitivity data, these 18 patients were older (median age 41 versus 36 years, p = 0.01) and had lower median CD4 counts at baseline (46/µL versus 80/µL, p = 0.04).

## Discussion

We report the prevalence of TB resistance to any anti-TB agent to be 7.4% (95% CI 4.0–12.4) among sputum culture positive patients initiating ART and intensively screened for TB in Durban, South Africa. Our data are consistent with the WHO South African estimate of 7.9% for any drug resistance among reported TB cases [13]. We found a high prevalence of rifampin resistance and MDR-TB, with a prevalence of 10.3% (2.9–24.2%) in patients previously treated for TB. MDR-TB has been associated with poor short-term outcomes among HIV-infected people in South Africa [14]. The scale-up of Xpert MTB/RIF screening in South Africa will allow for a more expedient diagnosis of rifampin-resistant TB and may improve TB outcomes by shortening diagnostic delays and ineffective initial therapy [15].

As observed in Tugela Ferry, a substantial proportion of patients with no prior history of TB had evidence of drug resistance. Of 13 patients with drug resistant TB, 5 (38%) had no prior history of TB and were not on TB treatment at enrollment. While prior TB treatment data are based on self-report, these results are consistent with data from Tugela Ferry showing that HIV-infected patients not previously treated for TB are at risk for exogenous infection with drug-resistant mycobacteria (primary resistance) [16]. In addition, we found high rates of streptomycin resistance among patients with a prior history of TB 10.3% (95% CI 2.9–24.2), calling into question the currently recommended TB retreatment strategy of adding streptomycin, a single agent, to the standard 4-drug TB induction regimen. [17], [18].

No cases of XDR-TB were found during the study period. This is in contrast to the high rates of XDR-TB seen among HIV-infected patients in the Msinga District (Tugela Ferry), also in the province of KwaZulu-Natal [7]. Despite close proximity between Tugela Ferry and Durban, there were differences between the site (rural provincial hospital versus semi-private urban hospital) and study population (inpatient and ambulatory versus ambulatory only). To what degree such environmental disparities or other treatment-related factors explain the difference in prevalence is uncertain.

There are several limitations to this study. Because McCord Hospital is a semi-private institution, patients presenting for treatment have the capacity to pay a small co-payment fee for the care received; the study sample may not be fully generalizable to the larger South African population. They may differ from those who receive care free of charge at governmental Department of Health clinics. Our study also focused solely on ambulatory patients. It is possible that inpatients at McCord Hospital have different resistance patterns than were observed here. However, because patients reported their previous 5 years of hospitalizations, where exposure rates are high, we believe that the patterns would not be markedly different. We do not have information regarding prior TB treatment regimens or completion. We are likely to have underestimated the overall prevalence of TB, as well as drug-resistant TB, because of the use of a single sputum culture for pulmonary TB diagnosis [12]. Additionally, not all sputum cultures had full sensitivity data available; our results may underestimate the true prevalence of drug-resistance.

Given the potential for acquisition of primary TB drug resistance among HIV-infected patients and worse outcomes for those infected with drug-resistant TB, this study argues in favor of improving infection control measures in the healthcare setting to minimize transmission of TB. Such measures, as well widespread implementation of rapid and accurate tools for diagnosing both TB and drug resistance in areas of high HIV/TB prevalence, are urgently needed.
